# An Account of the Beneficial Effects of the Flos Cerevisiæ, in a Case of Typhus Fever

**Published:** 1800-06

**Authors:** Charles Brown

**Affiliations:** Hatton Garden


### *An Account of the beneficial Effects of the Flos Cereviſiæ, in a Caſe of Typhus Fever.*

*By* CHARLES BROWN, *Surgeon.*

To the EDITORS of the MEDICAL and PHYSICAL JOURNAL.

Gentlemen,

READING in your valuable miſcellany, ſome obſervations on the medicinal uſe of yeaſt, by Mr. J. H. Groſe, I take the liberty of ſending you the particulars of a caſe of recovery, apparently from the exhibition of this valuable remedy.

Mrs. Northmore, living at Mr. Cadwallader's, in Charles Street, Hatton Garden, was delivered on the 25th of laſt December of a very fine boy.

The next day ſhe was as well as women uſually are in her ſituation; being coſtive, an opening mixture was preſcribed, which had the deſired effect. For three days ſubſequent to her lying-in, nothing particularly occurred, but on the fourth ſhe complained of pain in her limbs and cheſt, with head-ache, great thirſt and dryneſs about the fauces. Sudorific medicines were preſcribed; the next day ſhe was much better, when I preſcribed a weak decoction of the bark and ſnake-root. She was ſo much better on the 3d of January, as to be able to get out of bed, and ſit up for three hours. On the 6th, ſhe found herſelf again very poorly, and in the evening I was called in. I found her with a quick ſmall pulſe, pain in her head and limbs, and great proſtration of ſtrength; ſhe had been all the day very ſick at her ſtomach, and troubled with a griping pain in the abdomen. I preſcribed an emetic, and at bed-time a doſe of calomel with an opiate, which had their deſired effects. Through the whole of the next day, ſhe had a conſiderable remiſſion of the fever. On the 11th ſhe was much worſe. Her ſenſes were impaired, her pulſe very ſmall and quick, and ſhe had ſeveral ſtrong convulſive fits in the courſe of the day. At night ſhe had ſubſultus tendinum, and talked incoherently during the night. Upon viſiting her next day, I found her lying in a comatoſe ſtate. Her tongue and fauces were blackly furred, and her pulſe ſmall and quick. I directed a bliſter to be applied between the ſhoulders, and a ſaline draught with gr. viij of the antim. calcinat. to be adminiſtered every four hours. An opiate was given her at bed-time. She had a tolerable good night. Dr. Squire, a very able and humane practitioner, was conſulted next day, and the reſult was, to adminiſter the bark and port-wine very freely. Upon viſiting her the next day, we found her ſo ill, that it was the opinion of Dr. Squire and myſelf, ſhe could not poſſibly live twenty-four hours.—The bark ſhe rejected, nor would ſhe ſwallow any more wine, of which hitherto ſhe had taken plentifully. Quite deſpairing of being able to afford her any relief, and having prepared the minds of her friends for a ſpeedy diſſolution, I no longer placed any hopes on medicine. I ſent for a pint of freſh *yeaſt*, of which I directed a table ſpoonful to be given her every three hours, which directions were very punctually attended to.—A very unexpected alteration took place.—Her pulſe became quick, and leſs feeble; ſhe looked upon her friends about her, ſeemingly as if ſenſible ſhe was better—the ſubſultus tendinum ceaſed, and her ſkin became moiſt. The yeaſt was continued for ſeveral days, *ſhe* gradually mending; and after taking two quarts, was quite recovered.

*Hatton Garden,*

*April 3,* 1800.

I have the honour to be, &c.

